# Rectal stenosis due to solitary pelvic recurrence of hilar cholangiocarcinoma

**DOI:** 10.1002/jgh3.12357

**Published:** 2020-05-16

**Authors:** Ikuma Shioi, Yusuke Yamaoka, Akio Shiomi, Hiroyasu Kagawa, Hitoshi Hino, Shoichi Manabe, Daisuke Aizawa

**Affiliations:** ^1^ Division of Colon and Rectal Surgery Shizuoka Cancer Center Sunto‐gun Japan; ^2^ Division of Pathology Shizuoka Cancer Center Sunto‐gun Japan

## Abstract

An 85‐year‐old woman was admitted to a hospital with abdominal pain. Five years prior to admission, she had a history of hilar cholangiocarcinoma of pStage IIIC. Contrast‐enhanced computed tomography showed a mass between the rectum and uterus as well as bowel obstruction due to the lesion. Colonoscopy showed severe stenosis at the lower rectum and elevation of the submucosal layer with linear erosion. Rectal cancer was suspected, and pelvic recurrence of hilar cholangiocarcinoma or endometrial carcinoma infiltrating the rectum was considered as differential diagnosis. She underwent robot‐assisted low anterior resection combined with partial resection of the uterus. The immunohistopathological findings of the resected specimen favored a diagnosis of metastasis of cholangiocarcinoma, rather than primary rectal cancer or endometrial carcinoma. There were no signs of recurrence after 10 months of follow‐up. Hilar cholangiocarcinoma is a disease with poor prognosis. Recurrence is frequently experienced even after curative resection. Patients with recurrence are rarely candidates for re‐resection. However, better prognosis is reported for those with complete resection.

## Graphical abstract

Hilar cholangiocarcinoma is a disease with poor prognosis; recurrences are frequently experienced even after curative resection. However, very few reports focus on resection of the recurrence site. We herein report a unique case of rectal stenosis due to solitary pelvic recurrence of cholangiocarcinoma which was successfully resected.

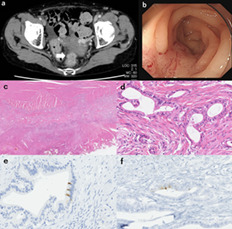

## Introduction

Hilar cholangiocarcinoma is a disease with poor prognosis. Recurrence is frequently experienced even after curative resection. Patients with recurrence are rarely candidates for reresection, but better prognosis is reported for those with complete resection. Here, we here report a unique case of an 85‐year‐old woman with rectal stenosis due to solitary pelvic recurrence of hilar cholangiocarcinoma, which was successfully resected. The patient remained free from cancer after 10 months of follow‐up.

## Case presentation

**Figure 1:**
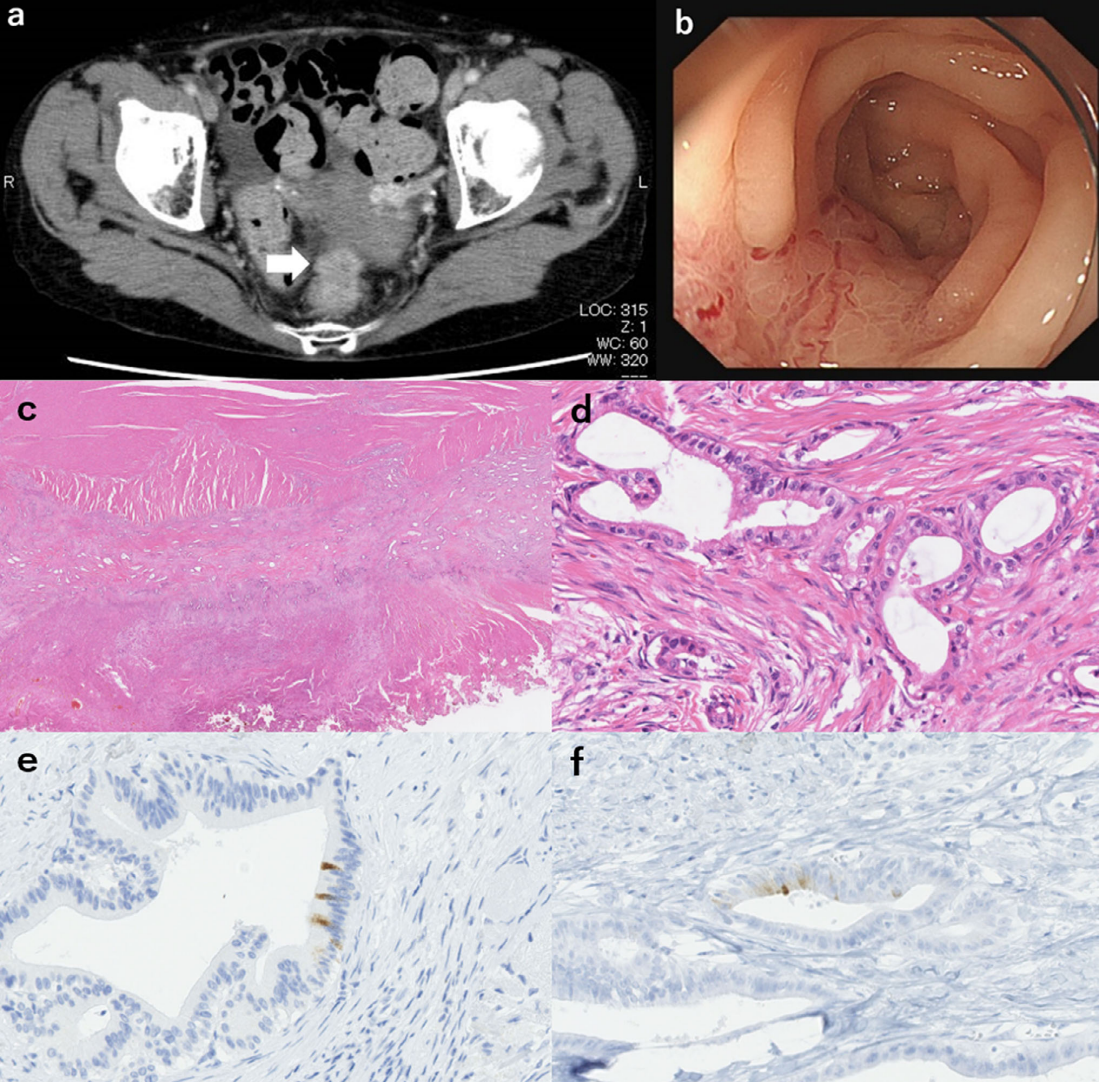
(a) Contrast‐enhanced computed tomography showed a mass between the lower rectum and uterus (arrow). (b) Colonoscopy revealed stenosis of the lower rectum with elevation of the submucosal layer and linear erosion. (c, d) Histopathological and immunohistochemistry results: Well‐differentiated tubular adenocarcinoma was observed. The lesion proliferated mainly at the outer layer of the rectal muscularis propria. Invasion of the uterus was observed. (c) Hematoxylin–eosin (HE) staining, ×100; (d) HE staining, ×400. (e, f) Most immunohistochemistry results were negative except for MUC6; (e) rectal specimen, MUC6, weakly positive, ×400; (f) cholangiocarcinoma specimen, MUC6, weakly positive, ×400.

An 85‐year‐old woman was admitted to a gastroenterology hospital with abdominal pain. Five years prior to admission, she had a history of left hepatic trisegmentectomy, caudal lobectomy, extrahepatic bile duct resection, and lymphadenectomy for hilar cholangiocarcinoma of pStage IIIC (pT3N1M0, according to the American Joint Committee on Cancer (AJCC) Cancer Staging Manual Eighth Edition^1^). As she did not wish for further treatment, she was under observation without any adjuvant therapy. There were no signs of locoregional nor hepatic recurrence on her follow‐up exam. On admission, a distended abdomen was noted on physical assessment. Contrast‐enhanced computed tomography (CT) showed a mass between the rectum and uterus, as well as bowel obstruction due to the lesion (Fig. 1a). Colonoscopy showed severe stenosis at the lower rectum and elevation of the submucosal layer with linear erosion (Fig. 1b). Histopathology of the colonoscopic biopsy specimen showed adenocarcinoma. A metallic stent was inserted, and her symptoms improved. Carcinoembryonic antigen and carbohydrate antigen 19–9 levels were within the normal range. Rectal cancer was suspected; however, the colonoscopy findings were not typical. Pelvic recurrence of hilar cholangiocarcinoma and endometrial carcinoma infiltrating the rectum was the considered differential diagnosis. Positron Emission Tomography CT was not conducted because the patient did not have any other lesions suspected to be metastasis. She underwent robot‐assisted low anterior resection combined with partial resection of the uterus. The histopathological findings of the resected specimen favored a diagnosis of well‐differentiated tubular adenocarcinoma. The lesion proliferated mainly at the outer layer of the rectal muscularis propria. Invasion of the uterus was observed. No intramucosal lesion was identified, which did not support a diagnosis of primary rectal cancer (Fig. 1c,d). Immunohistochemical staining was negative for cytokeratin 7 (CK7), CK20, mucin 2 (MUC2), MUC5AC, caudal‐related homeobox gene 2, CD10, and Estrogen receptor; it were weakly positive for MUC6 (Fig. 1e,f). These results were compared with the results obtained from former resected specimens; we found very close similarities in the staining patterns. The immunohistopathological profile was suggestive of metastasis of cholangiocarcinoma, rather than primary rectal cancer or endometrial carcinoma. Judging from the obtained results, she was diagnosed with solitary pelvic dissemination of hilar cholangiocarcinoma. She was under observation without any additional therapy, and there were no signs of recurrence after 10 months of follow‐up.

## Discussion

Cholangiocarcinoma is a disease with poor prognosis. Surgical resection with lymph node dissection is the only curative treatment. However, only 18–42% of patients are eligible for surgical treatment at the time of diagnosis.^2^ Even after curative resection, recurrence occurs in 50–75% of the patients,^3^, ^4^ which emphasizes the importance of treatment strategies after recurrence. The most common site of recurrence is the liver (34%), followed by locoregional recurrence (27%), lymph nodes (14%), and peritoneal dissemination (6%).^4^ Patients with recurrence are generally treated with chemotherapy,^5^ and their median survival time is reported to be 4.7–11.7 months.^5^, ^6^ Surgical resection of the recurrent lesion may prolong survival; however, curative resection can only be achieved in 13% of the patients.^3^ Noji *et al*.^7^ noted 27 cases of resected recurrent biliary carcinoma and reported that median survival time was 21.6 months, although it did not include cases with distant peritoneal dissemination. Miyata *et al*.^8^ reported a patient treated with multidisciplinary therapy who survived for more than 7 years after being diagnosed with peritoneal recurrence of hilar cholangiocarcinoma. Adjuvant therapies followed by surgery and chemotherapy alone or in combination with radiation therapy may prolong survival.^9^ However, in our case, as the patient did not wish for further treatment, she was under observation without any adjuvant therapy.

In our case, histopathological results favor dissemination of cholangiocarcinoma, which is usually observed in terminal‐stage patients as they have unresectable multiple lesions. However, it was successfully resected owing to its solitary state. To the best of our knowledge, there are no reports for solitary pelvic dissemination of cholangiocarcinoma, and this is the first report describing a curative resection of solitary pelvic recurrence of hilar cholangiocarcinoma.

## Declaration of conflict of interest

The authors declare no conflicts of interest associated with this manuscript.
